# Genetic Diversity and Distribution Patterns of Host Insects of Caterpillar Fungus *Ophiocordyceps sinensis* in the Qinghai-Tibet Plateau

**DOI:** 10.1371/journal.pone.0092293

**Published:** 2014-03-25

**Authors:** Qing-Mei Quan, Ling-Ling Chen, Xi Wang, Shan Li, Xiao-Ling Yang, Yun-Guo Zhu, Mu Wang, Zhou Cheng

**Affiliations:** 1 School of Life Science and Technology, Tongji University, Shanghai, China; 2 School of Plant Sciences and Technology, Agriculture and Animal Husbandry College of Tibet, Nyingchi, Tibet, China; University of Sydney, Australia

## Abstract

The caterpillar fungus *Ophiocordyceps sinensis* is one of the most valuable medicinal fungi in the world, and it requires host insects in family Hepialidae (Lepidoptera) to complete its life cycle. However, the genetic diversity and phylogeographic structures of the host insects remain to be explored. We analyzed the genetic diversity and temporal and spatial distribution patterns of genetic variation of the host insects throughout the *O. sinensis* distribution. Abundant haplotype and nucleotide diversity mainly existed in the areas of Nyingchi, ShangriLa, and around the edge of the Qinghai-Tibet Plateau, where are considered as the diversity center or micro-refuges of the host insects of *O. sinensis*. However, there was little genetic variation among host insects from 72.1% of all populations, indicating that the host species composition might be relatively simple in large-scale *O. sinensis* populations. All host insects are monophyletic except for those from four *O. sinensis* populations around Qinghai Lake. Significant phylogeographic structure (N_ST_>G_ST_, *P*<0.05) was revealed for the monophyletic host insects, and the three major phylogenetic groups corresponded with specific geographical areas. The divergence of most host insects was estimated to have occurred at ca. 3.7 Ma, shortly before the rapid uplift of the QTP. The geographical distribution and star-like network of the haplotypes implied that most host insects were derived from the relicts of a once-widespread host that subsequently became fragmented. Neutrality tests, mismatch distribution analysis, and expansion time estimation confirmed that most host insects presented recent demographic expansions that began ca. 0.118 Ma in the late Pleistocene. Therefore, the genetic diversity and distribution of the present-day insects should be attributed to effects of the Qinghai-Tibet Plateau uplift and glacial advance/retreat cycles during the Quaternary ice age. These results provide valuable information to guide the protection and sustainable use of these host insects as well as *O. sinensis*.

## Introduction

*Ophiocordyceps sinensis* (Berk.) Sung, Sung, Hywel-Jones and Spatafora (Ascomycota: Ophiocordycipitaceae), a caterpillar fungus [1], has been used for centuries as a traditional Chinese medicine to treat asthma, bronchial/lung infection, and kidney disease [2]–[4]. The host insects of *O. sinensis* belong to the family Hepialidae [5]. The fungus infects their larvae and forms sclerotia within the insect's intact exoskeleton, enabling the fungus to withstand the winter [6]. In spring and early summer, using the host insect for nutrition, the fungal fruiting body grows from the head of the dead larva and emerges from the soil surface [7]. Therefore, the host insect is essential to the life cycle of *O. sinensis*.

*Ophiocordyceps sinensis* and its host insects are endemic to the Qinghai-Tibet Plateau (QTP) in western China, i.e., Qinghai, Tibet, Yunnan, Sichuan, and Gansu provinces, where they are mainly distributed in alpine meadows [8], [9]. This fungus is becoming endangered through both overexploitation because of its commercial value, and habitat degeneration in recent years. Its specific host insects are also potentially endangered [10]. However, inducing fruiting bodies of *O. sinensis* by artificial inoculation of *Hepialus* larvae is still extremely challenging. Therefore, there exists an urgent need to establish appropriate strategies/policies to protect the endangered *O. sinensis* and its host insects.

Understanding the genetic diversity and phylogeographic structure of the host insects will provide important information for their conservation, as well as that of *O. sinensis*. Previous studies have focused mainly on the morphology, ecology, and life history of this interaction and reported that the host insects of *O. sinensis* are diverse and complex [6], [11]–[14]. Fifty-seven species representing seven genera (*Hepialus, Thitarodes, Pharmacis, Magnificus, Gazoryctra, Endoclita*, and *Bipectilus*) were considered to be possible hosts of *O. sinensis* [6]. Additionally, the most recent revision of the genus *Hepialus*, which has been adopted in China, divides the group into four genera (*Parahepialus gen*. nov., *Ahamus gen*. nov., *Hepialus*, and *Thitarodes*) [15]. Until now, few studies on the genetic relationships, population structure, and geographical distribution of the host insects have been reported, which significantly hinders the sustainable use and conservation of *O. sinensis* and its host.

The landscape features and climate patterns of southwest and northwest China were largely re-shaped by the uplift of the QTP [16], [17]. Many reports suggested that the unique complex topography and climate changes resulting from this rapid uplift explain the genetic divergence and speciation of some plants in the region [18]–[20]. The alternation of glacial and interglacial periods during the Quaternary also resulted in some species ranges retreating and expanding in the QTP [21], [22]. Additionally, insects are excellent indicators of endemism, as gene flow can be nearly completely interrupted by even weak habitat barriers in many species [23].

The host insects of *O. sinensis* show complex vertical and regional distribution patterns in the QTP [11], [13]. Different host species usually occupy different mountain ranges or even different sides and/or altitudes of the same mountain [11]. Phylogenetic analyses of mitochondrial Cyt*b* sequences from those host insects demonstrated that there exist many species with different geographical distributions [24]. Therefore, the distribution and species complexity of host insects may also have been influenced by the geological events and climatic shifts of the QTP. However, the distribution patterns and evolutionary histories of the host insects are still not clear.

The objectives of this study were to (i) analyze the genetic diversity and phylogeographic structure of the host insects using mitochondrial cytochrome oxidase subunit I (COI) sequences in large-scale *O. sinensis* populations covering the entire QTP; (ii) explore temporal and spatial distribution patterns of genetic variation in the host insects; (iii) infer what caused the spatial distribution pattern by estimating differentiation times and analyzing historical events in the QTP; and (iv) estimate possible historical population expansions and the evolutionary history of the host insects. Understanding the center of genetic diversity and possible causes of the current distribution patterns of these insects could provide valuable information to protect and sustainably use this host insect-*O. sinensis* system.

## Methods

### Ethics statement

No special permits were required for locations and samplings in this study. All samples were collected by researchers with introduction letters of IBAT (Institute of Bioresources and Applied Technology), Tongji University. The collection was completed with the help of local herdsman.

### Sampling

**Figure 1:**
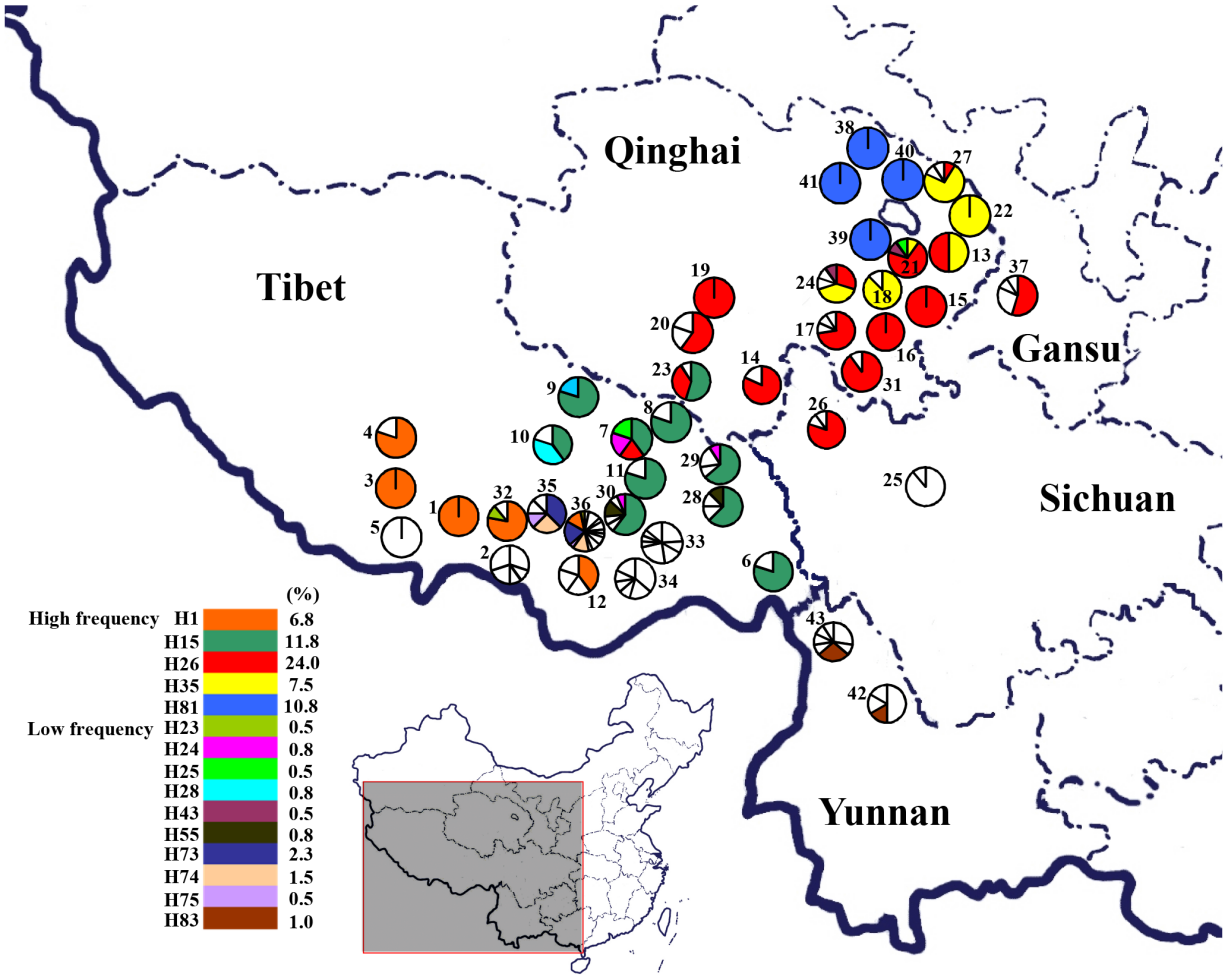
Geographical distribution and haplotype frequencies of host insects from 43 *O. sinensis* populations in the QTP. Population codes correspond to those in Table S1. Haplotype frequencies in each population are shown in the pie charts. The haplotypes existed in more than one population are color-coded, while private haplotypes are always shown by white circles.

A total of 400 samples from 43 *O. sinensis* populations, including 18 from Qinghai Province, 19 from Tibet, two from Gansu, two from Sichuan, and two from Yunnan, covered almost the entire distribution range in western China (Fig. 1; Table S1). These samples were complexes of *O. sinensis* stroma and host cadaver. In each population, we collected 5–30 samples that were at least 100 m apart. Collected specimens were preserved in the Herbarium of IBAT (Institute of Bioresources and Applied Technology), Tongji University, Shanghai, China. The codes, locations, and sample numbers of *O. sinensis* populations are shown in Table S1.

### DNA extraction, amplification, and sequencing

Total DNA was extracted from the heads of host cadaver using the CTAB method described previously [24]. The mitochondrial COI sequence is not only more reliable than either cytochrome oxidase subunit II (COII) or cytochrome *b* (Cyt*b*), but in combined analysis of COI+COII+Cyt*b* is also more representative of the phylogenetic relationships and geographical distribution patterns of host insects from different *O. sinensis* populations [25]. The host insect COI sequences of all 400 samples were sequenced. PCR were performed using the primers and protocols described by Hebert *et al.* [26]. Purification and sequencing were conducted by Shanghai GeneCore Biotechnologies Company, Shanghai, China.

### Molecular diversity

COI sequences were aligned using Mega 5.2 [27]. Molecular diversity indices, including the number of haplotypes (Nh), haplotype frequencies, number of private haplotypes (NpH), haplotype diversity (Hd), and nucleotide diversity (Pi), were calculated using DnaSP version 5.1 [28] for each population.

### Phylogenetic analysis and haplotype network

Haplotype phylogenetic relationships were reconstructed using three optimality criteria: Bayesian inference (BI), maximum likelihood (ML) and Neighbour-Joining (NJ). MrModel test2 v.2.3 [29] in conjunction with PAUP 4.0b10 [30] was used to select the best model for the BI and ML analyses. BI and ML analyses implemented the GTR+I+G model for each data partition. BI analysis with Markov chain Monte Carlo (MCMC) method was conducted in MRBAYES 3.1.2 [31], the Markov chains were run for 10 000 000 generations with trees being sampled every 100 generations. The first 25 000 generations were discarded as burn-in, and the remaining trees were used to estimate Bayesian posterior probabilities. ML analysis was conducted using PhyML 3.0 [32]. NJ analysis was implemented using Mega 5.2 [27], the bootstrap values for the interior nodes in the NJ tree were performed with 1000 replicates.

The COI sequences of four Hepialidae species, *Ahamus yushuensis* (HM595854, HM595849, HM595840 and HM595850), *Hepialus menyuanicus* (HM595858), *Thitarodes renzhiensis* (HM744694) and *Thitarodes armoricanus* (HM595856) obtained from GenBank were used to evaluate the possible species of the host insects of *O. sinensis* in this study. *Papilio* spp. (JQ606303) was selected as an outgroup taxon to root the phylogenetic trees. Genealogical relationships among the observed haplotypes in each defined group were examined using Network 4.6 [33]. These defined groups in the phylogenetic trees were detected whether they belong to monophyly using the species delimitation plugin [34] for Geneious Pro v4.8.4 [35].

### Analysis of genetic differentiation and phylogeographic structure

Phylogeographic structure was tested by calculating two measures of genetic differentiation: G_ST_ and N_ST_ [36], [37]. G_ST_ considers only haplotype frequencies while N_ST_ also takes into account differences among haplotypes. A higher N_ST_ than G_ST_ usually indicates the presence of phylogeographic structure [36], [37]. Therefore, the two parameters were compared using a permutation test with 1000 permutations and the *U*-statistic as implemented in PERMUT (available at http://www.pierroton.inra.fr/genetics/labo/Software/Permut). Analysis of molecular variance (AMOVA) was performed to distinguish variation within and among populations using Arlequin 3.11 [38], with significance tested via 1000 nonparametric permutations. These analyses were calculated for the monophyletic host insects and also for its each genetic group individually.

### Demographic history analyses

Fu's *Fs*, Fu and Li's D* and F*, and Tajima's *D* tests of neutrality were calculated to detect the range of population expansions [39]–[41]. These parameters provide information about demographic histories; significant negative values can be taken as evidence of demographic expansions, while positive values might result from population bottlenecks. Mismatch distribution analysis was conducted to further infer demographic processes using Dnasp5.1 [28]. A unimodal distribution indicates that populations have passed through a recent demographic expansion [42], while multimodal distributions are consistent with stability [43].

### Divergence time estimates

Because Hepialidae fossils are lacking, divergence time among haplotypes in this study were estimated assuming the substitution rate of 1.02% Myr^−1^ for *Papilio* spp. for COI+COII [44]. This estimation was performed using program Mega 5.2 [27] on a Neighbor-Joining tree with a topology similar to the BI and ML trees, in order to avoid the effect of model and arithmetic of different software on divergence time.

Expansion time was estimated using the expectation τ = 2*ut* [45], where tau (τ) is the age of expansion in mutational units derived from the mismatch distribution, *t* is the absolute time since expansion, and *u* = 2*μk*, where *μ* is the mutation rate and *k* is the length of the sequence [46]. We assumed a generation time of 3.5 years, because *Hepialus* generations last 3–4 years in alpine meadow environments [11].

## Results

### Haplotype diversity

There were no insertions or deletions of nucleotides in the 654 bp COI sequences amplified from 400 samples from all 43 *O. sinensis* populations. A total of 169 nucleotide polymorphisms were detected. The total haplotype diversity (Hd) and nucleotide diversity (Pi) of COI were 0.908 and 0.041, respectively. Within populations, host Hd was in the range of 0–0.924 and Pi was in the range of 0–0.058 (Table S1). Population P36, located in the Nyingchi area of Tibet, showed the highest haplotype and nucleotide diversity (Fig. 1, Table S1). Other populations from the Nyingchi and ShangriLa areas also had high haplotype diversity.

Ninety-one distinct haplotypes of COI were identified in host insects from the 43 populations (Table S2, GenBank accession No. KC994913-KC995003). Fifteen haplotypes were shared among multiple populations with obvious geographical distribution, but only five had frequencies above 5%, including H1 (6.8%), H15 (11.8%), H26 (24.0%), H35 (7.5%), and H81 (10.8%) (Fig. 1). These five haplotypes were detected in 60.9% of all samples and in 81.4% of all populations; they had a broad distribution in the QTP but were mainly distributed, respectively, to the west of the Nyingchi area, the north of the Nyingchi area, in the mid-south of Qinghai, in northern Qinghai, and in the area around Qinghai Lake (Fig. 1). Some shared haplotypes were unique to populations from specific regions, e.g., the ShangriLa or Qinghai Lake areas. The other 76 private haplotypes were detected in 29 populations (67.4% of all populations). Interestingly, only five populations (P2, P5, P25, P33, and P34) lacked shared haplotypes, and they were usually located along the edge of the distribution areas.

### Phylogenetic relationships and host haplotype network

**Figure 2:**
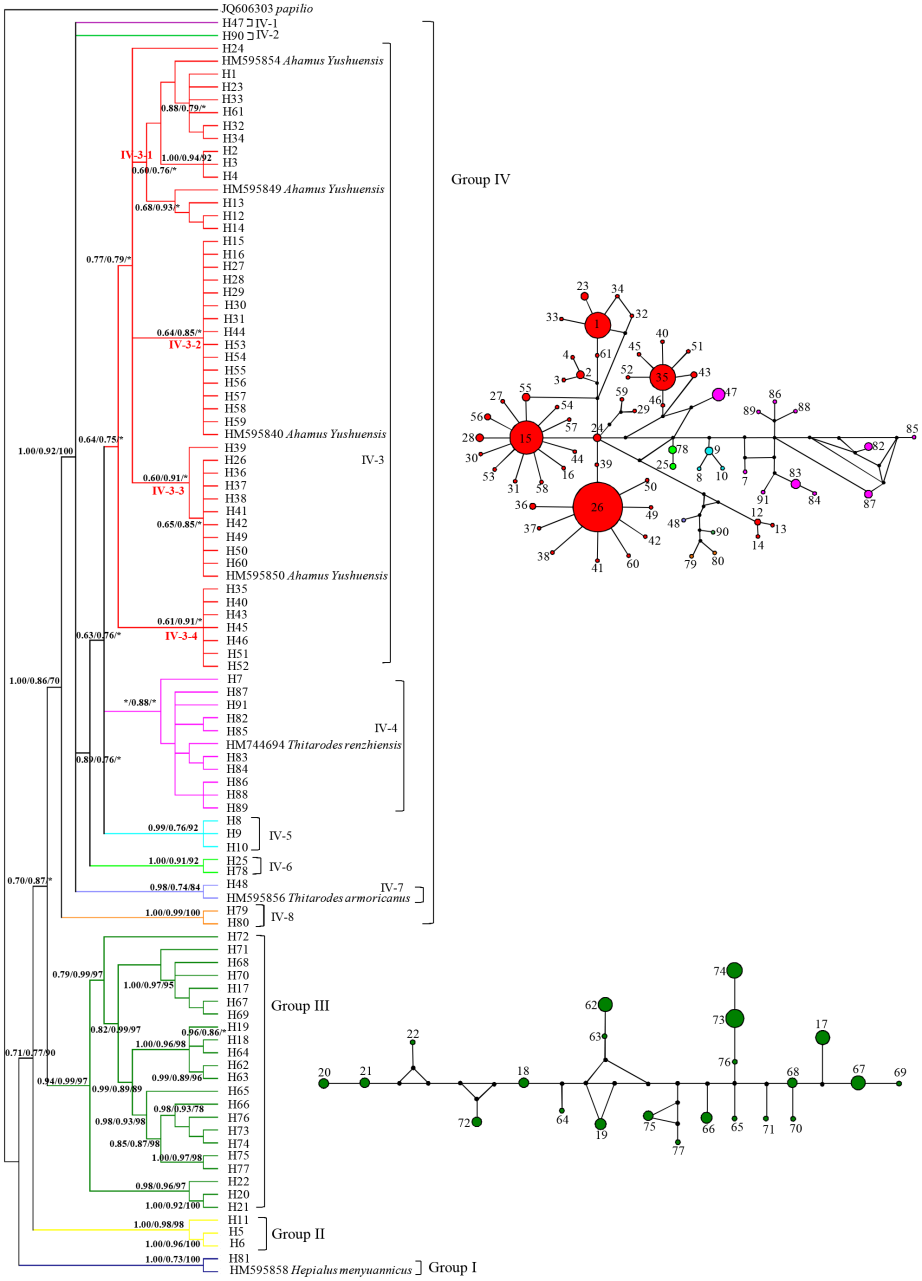
Phylogenetic relationships and haplotypes network of host insects from 43 *O. sinensis* populations in the QTP. (Left) Bayesian haplotypes phylogenetic tree of host insects based on COI gene sequences. Numbers on branches are Bayesian posterior probabilities, bootstrap support values of Maximum likehood and Neighbour-Joining trees (BI/ML/NJ), and * represented posterior probabilities <0.6 or support values <60%. The black bars on the right indicate the corresponding group number in the nested clade phylogeographic analysis, and each group or subgroup was presented with different colors. (Right) Haplotype networks of host insects corresponding to the group III and IV. The relative sizes of circles in the network are proportional to haplotype frequencies. The small black dots indicate hypothetical missing haplotypes. The haplotype colors correspond to those in phylogenetic tree on the left.

In the Bayesian tree of host COI sequences, four haplotype groups were distinguished, which was largely congruent with that of NJ and ML trees differing only in minor rearrangements of the leaves (Fig. 2). Monophyletic analysis revealed that all host insects were monophyletic except those in group I. Group I included only haplotype H81, the sole haplotype in four populations around Qinghai Lake, which clustered to *Hepialus menyuanicus* with high posterior probability or bootstrap value (PP/ML/NJ = 1.00/0.73/100).

Group II comprised the private haplotypes H5, H6, and H11 and included the two populations P2 and P5, which were located on the edge of southern Tibet. Group III included haplotypes H17-H22 and H62-H77, which were restricted to populations of P33, P34, P35, and P36 from the Nyingchi area. The remaining 71.4% of haplotypes were clustered into group IV, which could be subdivided into eight subgroups (Fig. 2). The subgroup IV-3 with the most haplotypes was clustered with *Ahamus yushuensis*, and further divided into IV-3-1, IV-3-2, IV-3-3, and IV-3-4, corresponding to the four dominant haplotypes H1, H15, H26, and H35, respectively (Fig. 2). The other seven subgroups were mainly composed of haplotypes from populations P4, P25, P37, P42, and P43 with high posterior probability or bootstrap values, which were located on the edge of the host insect distribution. The subgroup of IV-4 and IV-8 clustered to the *Thitarodes renzhiensis* and *T. armoricanus*, respectively.

No haplotype network was calculated for groups I and II, which had few haplotypes. The haplotype network for group III, which contained only haplotypes fixed in those populations from the Nyingchi area, showed no clear indication of demographic processes (Fig. 2). Four populations (9.3% of the total) from the Nyingchi area possessed 34.2% of all private haplotypes. The large number of haplotypes suggested that the Nyingchi area was a diversity center for the host insects of *O. sinensis*. The network for group IV showed that a large number of private haplotypes were connected in a star-like manner, with some dominant haplotypes such as H1, H15, H26, and H35 (Fig. 2). Haplotype H24 was considered as the central haplotype in this network. H24 had only four, one, two, and four mutations different from the four domain haplotypes of H1, H15, H26, and H35, respectively. The dominant haplotype H1 occurred mainly in populations located to the west of the Nyingchi area. H15 occurred in eight populations located to the north of the Nyingchi area and also appeared in populations P23 from southern Qinghai and P6 from the Nyingchi area. H26 was the most widespread haplotype and existed in populations from mid-south Qinghai, Gansu, and Sichuan Provinces. The two haplotypes H15 and H26 with the highest distribution frequencies simultaneously appeared in the populations P7 and its closer population P23. In particular, population P7 also contained the central haplotype H24 (Fig. 1). The dominant haplotype H35 was distributed in the populations from north Qinghai and usually appeared together with H26. The distal haplotypes positioned away from the four haplotypes had complex connections (Fig. 2). They were usually found in populations P25, P37, P42 and P43, which were located on the eastern edge of the distribution area (Fig. 1).

### Genetic differentiation and phylogeographic structure

AMOVA analysis revealed that 65.18% of the total genetic variation was partitioned among populations, indicating remarkable genetic differentiation in the monophyletic host insects (Table 1). Different genetic differentiation patterns appeared in different groups (Fig. 2). Group II had the highest percentage of genetic variation (96.8%) among populations, indicating that extreme differences existed between the two populations (P2 and P5) in this group. High levels of genetic variation (59.28%) existed among populations in group IV, especially in subgroup IV-3 (75.56%), which included most populations with broad distribution ranges in western China, and could reflect the fundamental genetic differentiation of these host insects. However, group III showed a peculiar genetic differentiation pattern in that only 38.51% of the genetic variation occurred among populations (Table 1), unlike in the other groups or in all populations.

**Table 1:** Estimates of genetic differentiation coefficient (N_ST_, G_ST_) and analysis of molecular variance (F_ST_) for host insects from 43 *O. sinensis* populations in the QTP.

	N_ST_	G_ST_	F_ST_
Monophyly	0.7498	0.3990	0.6518
Group II	0.9680	0.5380	0.9680
Group III	0.4370	0.1000	0.3851
Group IV	0.6340	0.4380	0.5928
Subgroup IV-3	0.8036	0.4899	0.7556

Monophyly (Group II+Group III+Group IV) was detected using Geneious Pro v4.8.4 software. Each group and subgroup was defined according to BI analysis and corresponding to the phylogenetic tree in Figure 2.

A permutation test showed that N_ST_ (0.7498) was significantly higher than G_ST_ (0.3990, *P*<0.05) for the monophyletic host insects, indicating that significant phylogeographic structure existed in the host insects of *O. sinensis*. The phylogeographic structure was also significant for each group.

### Divergence times and demographic expansion

Divergence time estimation revealed that the initial divergence of host insects occurred around 4.1 Ma, in the late Miocene. Host haplotypes nested within group II diverged more recently, around 2.2 Ma, in the Pleistocene. Haplotype diversification of groups III and IV began almost simultaneously around 3.5 Ma and 3.7 Ma, respectively, in the late Pliocene.

**Figure 3:**
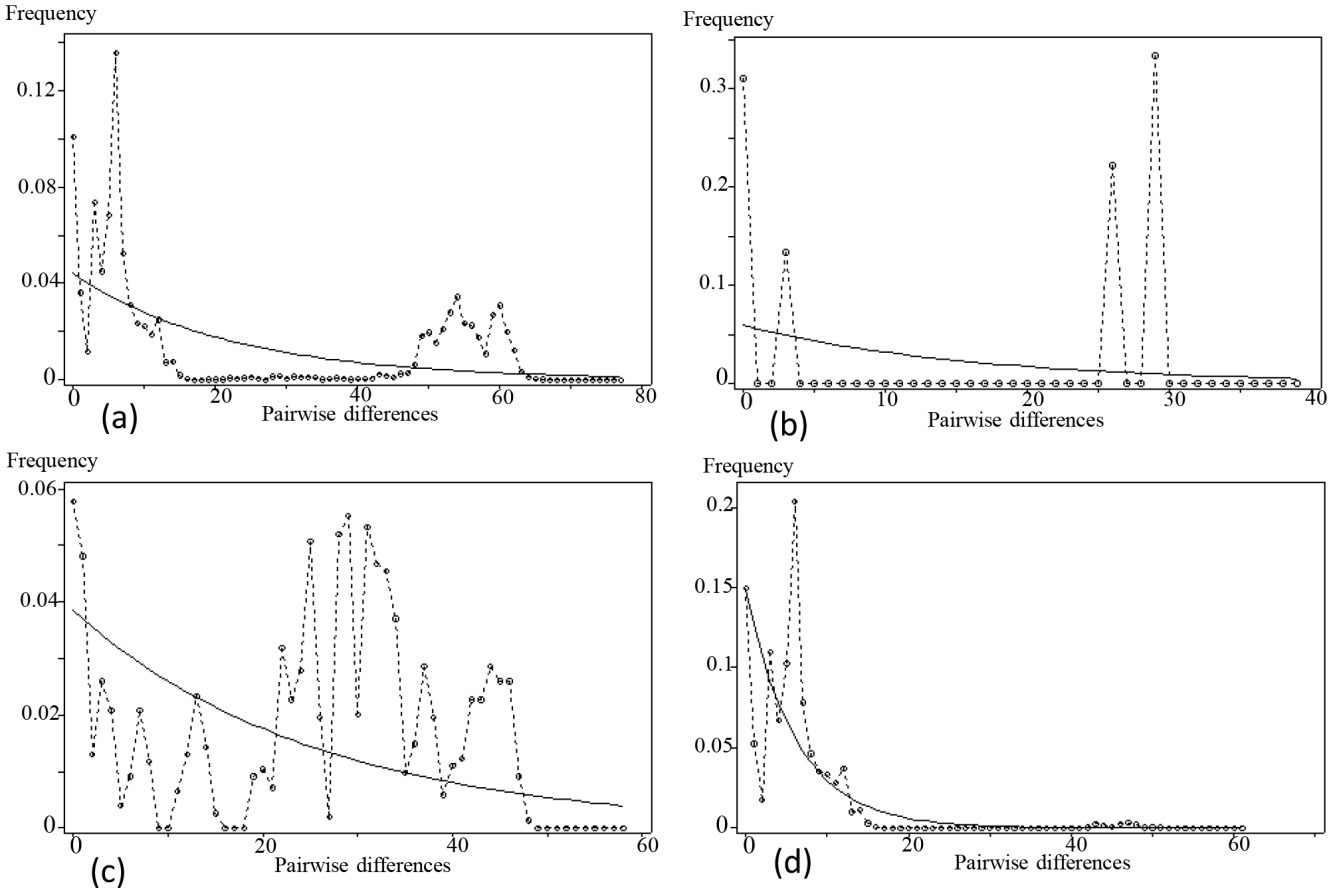
Mismatch distribution analysis for host insects using DNASP5.1. Graphs of the mismatch distributions of (a) Monophyly; (b) group II; (c) group III; (d) group IV. The X axis shows the observed distribution of pairwise nucleotide differences, and the Y axis shows the frequencies. The dotted lines with circles represent the observed frequency of pairwise differences, and the solid lines show the expected values under the sudden population expansion model.

Three neutrality tests resulted in non-significant negative values for all monophyletic host insects (Table 2), and mismatch distribution analysis showed a multimodal curve (Fig. 3a). These analyses indicated that no obvious expansion had occurred in the monophyly. However, different demographic processes occurred in different groups (Fig. 3). In group III, an expansion model was rejected based on non-significant positive values of the neutrality tests and on the multimodal curve (Fig. 3c). However, significant positive values of the neutrality tests and a multimodal curve were obtained from group II (Fig. 3b), indicating maybe a recent population bottleneck in this group. Conversely, Fu's *Fs*, Fu and Li's F* and Tajima's *D* were all negative and significant for group IV, demonstrating that a recent demographic expansion was the likely cause of the deviances from neutrality (Table 2). The unimodal curve of the mismatch distribution analysis agreed with the neutrality test results (Fig. 3d). The expansion time of group IV was estimated to have begun around 0.128 Ma, in the late Pleistocene. The expansion estimate of subgroup IV-3 was completely consistent with that of group IV, and its expansion time was also in the late Pleistocene, around 0.118 Ma.

**Table 2:** Estimates of the neutral tests (Fu's *F_S_*, Fu and Li's D*, Fu and Li's F*, and Tajima's D) for population expansion of each group.

	*Fs*	P value	D*	P value	F*	P value	Tajima's D	P value
Monophyly	−10.32	P>0.10	−0.65	P>0.10	−0.85	P>0.10	−0.79	P>0.10
Group II	10.64	P>0.05	1.59	P<0.02	2.09	P<0.02	2.62	P<0.01
Group III	4.80	P>0.05	0.56	P>0.10	0.50	P>0.10	0.19	P>0.10
Group IV	−24.78	P<0.01	−2.06	P>0.05	−2.37	P<0.05	−1.90	P<0.05

*Fs*, Fu's *Fs* test statistic; D *, Fu and Li's D * test statistic; F*, Fu and Li's F* test statistic; Tajima's *D*: Tajima's Test statistic.

## Discussion

Haplotype diversity of the host insects was very high (Hd = 0.908). Based on identified Hepialidae species and the COI haplotypes in this study, the host insects belong to three genera: *Ahamus*, *Hepialus* and *Thitarode*. The high haplotype diversity and apparent species composition support the idea that the host insects of *O. sinensis* are complex and belong to many species of different genera [6]. However, most host haplotypes were derived from dominant ones. Four dominant haplotypes with few nucleotide mutations represented 50.7% of samples and 72.1% of populations with a broad distribution in western China. The four dominant haplotypes clustered in subgroup IV-3 corresponded to *Ahamus yushuensis*. Our previous study using Cyt*b* sequences demonstrated that *A. yushuensis* was widely distributed, although most host species have narrow distributions [24]. Therefore, in this study, the insects from most populations were closely related, indicating that the host species composition might be relatively simple in large-scale *O. sinensis* populations.

In this study, all host insects were monophyletic except for those from four *O. sinensis* populations around Qinghai Lake (group I), in complete accordance with the results revealed by Cyt*b* sequences [24]. Qinghai Lake, the largest extant closed-basin lake in China, is surrounded by the Datong, Riyue, and Amuni mountains [47], [48]. The Qinghai Lake basin was fractured from the northwest to the southeast during the QTP uplift in the late Tertiary. During the middle Pleistocene, vast crustal movements caused the area to sink substantially, forming the lake [47]. The regional climate, rainfall, and plant distributions in this area were shaped by the water of the lake itself and by the topography of the basin [49]. Group I of the host insects included a sole private host haplotype (H81) whose ancestor was estimated to have occurred ca. 4.1 Ma, before the onset of the major Quaternary glaciations. Thus, the unique host insects of *O. sinensis* likely evolved under the special environment and topography of Qinghai Lake.

AMOVA analysis indicated that most genetic variation (F_ST_ = 65.18%) in the monophyletic host insects occurred among populations. High levels of genetic differentiation have also been found for numerous other alpine species in the QTP [50]–[53]. Distinct phylogeographic structure is reported to be usually coupled with high genetic differentiation among populations [54]. In this study, significant phylogeographic structure (N_ST_>G_ST_, P<0.05) did exist in the monophyletic host insects across the distribution of *O. sinensis*. Many factors have been suggested to influence the patterns of genetic and geographic structure in natural populations, such as life history, ecological traits, habitat, and historical events [55]–[58]. In this study, the significant phylogeographic structure of the host insects should be attributed to their unique life history and habitat isolation. Female *Hepialus* cannot fly long distances, and adults survive only 3–8 days [11]. The complex topography resulting from the uplift of the QTP [59] also limited gene flow among populations. Usually, significant phylogeographic structure demonstrates that the most closely-related haplotypes are more likely to co-occur in the same geographical area [50]. In this study, three haplotype groups of the monophyletic host insects corresponded with the edge of southern Tibet (group II), the Nyingchi area (group III), and the mid-south of Qinghai to mid-north of Tibet and related areas (group IV) (Fig. 2), indicating that the host insects had obvious geographical distribution patterns.

Both populations (P2 and P5) in group II and some populations (P25, P37, P42, and P43) in group III, which are located on the edge of the QTP, had unique haplotypes and high genetic diversity. Group II was estimated to have split ca. 2.2 Ma, probably because of the geographical and ecological alterations following both the QTP uplift and glacial advance/retreat cycles during the Quaternary ice age [60], [61]. The margin of the QTP, which had complex topography and a special ecoclimate, served as a refugium or source for many species [50], [52]. In particular, the southeastern margin of the QTP, the Hengduan Mountains region, not only served as an important glacial refugium but was also a center of diversification for many plants and animals [52], [62], [63], because extensive ice did not develop there during the Quaternary stage [64]. Populations P42 and P43 were located in the ShangriLa area of the Hengduan Mountains region. Therefore, we hypothesized the existence of multiple periglacial micro-refugia for the host insects around the edge of the QTP during glacial periods. This phenomenon is similar to results of phylogeographic analyses of other species in the QTP [50], [52], [65]. However, in this study, those edge refugia were not a source of private haplotypes spreading to other areas during inter- or post-glacial periods. This unique aspect may be related to the short flying distance and weak dispersal ability of the host insects of *O. sinensis* [11].

The highest host haplotype and nucleotide diversities were detected in group III, suggesting that the Nyingchi area was a diversity center or another refugium of the host insects of *O. sinensis* during the glacial period. The divergence of group III was estimated to have occurred ca. 3.5 Ma, following the changed monsoon intensity [61], [66], modified global climate [61], [67], [68] and topography changes caused by the rapid uplift of the QTP at ca. 3.6 Ma [60], [69]. Geological studies demonstrated that both uplift speed and amplitude of this area of southeastern Tibet were slower than in the western Himalayas and northern Tibet during the late Pleistocene [70]. According to a report from the China Tibet Information Center, a gap that opened in the Nyingchi area allowed the warm Indian current to move upstream to meet cold air from the north, producing different coexisting bioclimatic regions in the area (http://www.tibetinfor.com.cn/english/reports/land/nyingchi/nyingchi.htm). The haplotype network and neutrality tests demonstrated no evidence of postglacial recolonization or range expansion in this area. Additionally, the genetic differentiation within populations was relatively high compared with other areas. Therefore, the unique topography and climate of the Nyingchi area provided refuges and led to the diverse host haplotypes found today in Tibet.

Most host haplotypes from populations covering almost the entire QTP were included in group IV. Differentiation in this group was estimated to have occurred ca. 3.7 Ma, shortly before the rapid QTP uplift at ca. 3.6 Ma [60], [69]. The QTP uplift might play an important role in the divergence of host insects of *O. sinensis*. Four dominant host haplotypes (H1, H15, H26, and H35) corresponding to distinct areas distributed along a latitudinal gradient were defined based on the phylogenetic analysis and haplotype geographic distribution. This finding was congruent with the distribution pattern of *O. sinensis* based on morphological characteristics and ISSR analysis [71]. The four dominant haplotypes in subgroup IV-3 corresponded to only *A. yushuensis*. There were only a few nucleotide differences among the four main haplotypes, but few populations had two or more of these haplotypes. Additionally, each dominant host haplotype usually only occupied a single distribution area. These results implied that the rapid uplift of the QTP led to habitat isolation and nucleotide mutations in the host insects. Many studies have indicated that habitat fragmentation could have a great influence on plant divergence and speciation [18], [72]–[74]. Therefore, we hypothesized that the haplotypes in this group were derived from relicts of a once widespread host insect species that subsequently became fragmented.

A recent demographic expansion in subgroup IV-3 (*A. yushuensis*) was confirmed by neutrality tests and mismatch distribution analysis. The star-like haplotype network, high haplotype diversity, and low nucleotide diversity also indicated that host insects in this subgroup had undergone rapid regional expansion. The expansions of host insects within this subgroup were estimated to have begun ca. 0.118 Ma, in the late Pleistocene, during the inter-glacial period after the penultimate glaciation (ca. 0.3–0.13 Ma) [75]–[78]. We inferred that an ancestral haplotype was widely distributed before the uplift occurred and, subsequently, geological changes and climatic oscillations during the QTP uplift and Quaternary glaciation led to the present-day genetic diversity and population differentiation of the host insects. After the QTP uplift and glaciation period, the four dominant haplotypes appeared in their respective latitudinal zones. Many new host haplotypes were derived from these four during these expansion phases, resulting in the star-like haplotype network that we recovered. Population P7 included two dominant haplotypes (H15, H26) as well as a central haplotype (H24) with only one nucleotide difference from H15, which may be situated at the point where several recolonizing lineages merged.

Conservation of rare species focuses mainly on maximizing genetic diversity and heterozygosity [79], because high genetic diversity can yield higher fitness during environmental changes [80]. In this study, the genetic diversity and structure we identified not only provided important insights into the evolutionary history of the host insects but these are also critical for their conservation management. Our results showed that the conservation of multiple populations in both the Nyingchi and ShangriLa areas will be necessary to ensure the genetic and species diversity of these insects. Additionally, the *O. sinensis* populations with high host genetic diversity (e.g., population P7) from the four latitudinal regions that cover most of the QTP should be given high priority for protection and sustainable development.

## Supporting Information

Table S1 **Codes, locations and sample numbers of** ***O. sinensis*** **populations and genetic diversity estimates of its host insects.** P: population; N: number of specimen; Nh: number of haplotypes; S: number of segregating sites; Hd: haplotype diversity; Pi: nucleotide diversity; NpH: number of private haplotypes.
(XLSX)

Table S2 **GenBank accession numbers and haplotype frequencies in the populations included in the study.** Population codes correspond to those in Table S1 and Figure 1.
(XLSX)
